# Supplementary material for: How many cyberbullying(s)? A non-unitary perspective for offensive online behaviours
Source: PLoS One. 2022 Jul 19;17(7):e0268838. doi: 10.1371/journal.pone.0268838 (PMC9295961; doi:10.1371/journal.pone.0268838)
Supplement: S1 Text — Full text of questionnaire used in the study. (PDF) [file pone.0268838.s001.pdf]

Supporting information 1: Full text of questionnaire used in the study

Sezione anagrafica

Età: ____________________

Genere: M / F

Tipo di scuola che frequenti:
- ○ Liceo Classico
- ○ Istituto Tecnico
- ○ Liceo Scientifico
- ○ Istituto Professionale
- ○ Liceo Artistico
- ○ Liceo della Formazione
- ○ Liceo Linguistico

Sezione uso social media

Quali dei seguenti social usi principalmente?
- ○ Tumblr
- ○ Google+
- ○ yahoo!
- ○ Answer
- ○ Youtube
- ○ ask.fm
- ○ Instagram
- ○ Facebook
- ○ Snapchat
- ○ Whatsapp
- ○ Twitter Pinterest

Quanto ti senti nervoso se non puoi usare Internet?
(Per niente) 0 - 1 - 2 - 3 - 4 (Molto)

Quanto tempo mediamente trascorri sul social che utilizzi di più?
- ○ < 1 ora/giorno
- ○ 1-3 ore al giorno
- ○ > 3 ore al giorno

Sui social hai
- ○ Nessun profilo
- ○ Un solo profilo
- ○ Più di un profilo

In TUTTO hai __________ follower

Sezione personalità

Mi vedo come una persona che

È riservata
(Per niente d'accordo) 1 - 2 - 3 - 4 - 5 (Del tutto d'accordo)

Generalmente si fida
(Per niente d'accordo) 1 - 2 - 3 - 4 - 5 (Del tutto d'accordo)

Tende a essere pigra
(Per niente d'accordo) 1 - 2 - 3 - 4 - 5 (Del tutto d'accordo)

È rilassata, sopporta bene lo stress
(Per niente d'accordo) 1 - 2 - 3 - 4 - 5 (Del tutto d'accordo)

Ha pochi interessi artistici
(Per niente d'accordo) 1 - 2 - 3 - 4 - 5 (Del tutto d'accordo)

È spigliata, socievole
(Per niente d'accordo) 1 - 2 - 3 - 4 - 5 (Del tutto d'accordo)

Tende a trovare i difetti negli altri
(Per niente d'accordo) 1 - 2 - 3 - 4 - 5 (Del tutto d'accordo)

È coscienziosa nel lavoro
(Per niente d'accordo) 1 - 2 - 3 - 4 - 5 (Del tutto d'accordo)

Si agita facilmente
(Per niente d'accordo) 1 - 2 - 3 - 4 - 5 (Del tutto d'accordo)

Ha una fervida immaginazione
(Per niente d'accordo) 1 - 2 - 3 - 4 - 5 (Del tutto d'accordo)

Sezione disimpegno morale

Cyberbullizzare compagni di classe fastidiosi è soltanto dare loro una lezione
(Per niente d'accordo) 1 - 2 - 3 - 4 - 5 (Del tutto d'accordo)

Ai ragazzi non importa davvero di essere cyberbullizati perché questo mostra che gli altri sono interessati
(Per niente d'accordo) 1 - 2 - 3 - 4 - 5 (Del tutto d'accordo)

Va bene trattare male qualcuno se si comporta da stupido
(Per niente d'accordo) 1 - 2 - 3 - 4 - 5 (Del tutto d'accordo)

Se le persone danno le loro password ad altri, meritano di essere cyberbullizzati
(Per niente d'accordo) 1 - 2 - 3 - 4 - 5 (Del tutto d'accordo)

Va bene vendicarsi se qualcuno fa il prepotente con uno dei tuoi amici
(Per niente d'accordo) 1 - 2 - 3 - 4 - 5 (Del tutto d'accordo)

Va bene diffondere cattivi pettegolezzi su qualcuno perché non è così grave come picchiarlo
(Per niente d'accordo) 1 - 2 - 3 - 4 - 5 (Del tutto d'accordo)

I ragazzi che cyberbullizzano gli altri ragazzi perché i loro amici li spingono a farlo non dovrebbero essere colpevolizzati per quello che fanno
(Per niente d'accordo) 1 - 2 - 3 - 4 - 5 (Del tutto d'accordo)

Se i ragazzi cyberbullizzano gli altri a scuola, è colpa dell'insegnante per non averli fermati
(Per niente d'accordo) 1 - 2 - 3 - 4 - 5 (Del tutto d'accordo)

Sezione cyberbullismo

Quante volte hai inviato messaggi cattivi o indecenti?
- Mai
- 1-2 volte
- 3-5 volte
- Più di 5 volte

Quanto spesso hai inviato email cattive o indecenti?
- Mai
- 1-2 volte
- 3-5 volte
- Più di 5 volte

Quanto spesso hai inviato foto imbarazzanti di qualcuno via cellulare?
- Mai
- 1-2 volte
- 3-5 volte
- Più di 5 volte

Quanto spesso hai finto di essere qualcun'altro su Internet?
- Mai
- 1-2 volte
- 3-5 volte
- Più di 5 volte

Quante volte ti è capitato di dire i segreti di qualcun'altro online o attraverso il cellulare senza permesso?
- Mai

- 1-2 volte
- 3-5 volte
- Più di 5 volte

Quanto spesso hai diffuso pettegolezzi riguardo qualcuno su Internet?

- Mai
- 1-2 volte
- 3-5 volte
- Più di 5 volte
