# Supplementary material for: How many cyberbullying(s)? A non-unitary perspective for offensive online behaviours
Source: PLoS One. 2022 Jul 19;17(7):e0268838. doi: 10.1371/journal.pone.0268838 (PMC9295961; doi:10.1371/journal.pone.0268838)
Supplement: S1 File — Each plot represents the estimated probability of the different responses concerning the frequency of perpetration of a given type of cyberbullying behaviour, as a function of one of the significant predictors. The probabilities were estimated from the fitted regression models presented in the paper. All the code for reproducing the plots is provided in S2 File. (PDF) [file pone.0268838.s008.pdf]

Supporting information 3: Plots of the effects of the significant predictors in the ordinal logistic regression models.

In the following pages are presented the effects plots for all the significant predictors in the ordinal regression models. Each plot represents the estimated probability of the different responses concerning the frequency of perpetration of a given type of cyberbullying behaviour, as a function of one of the significant predictors. The probabilities were estimated from the fitted regression models presented in the paper. All the code for reproducing the plots is provided as S2 File.

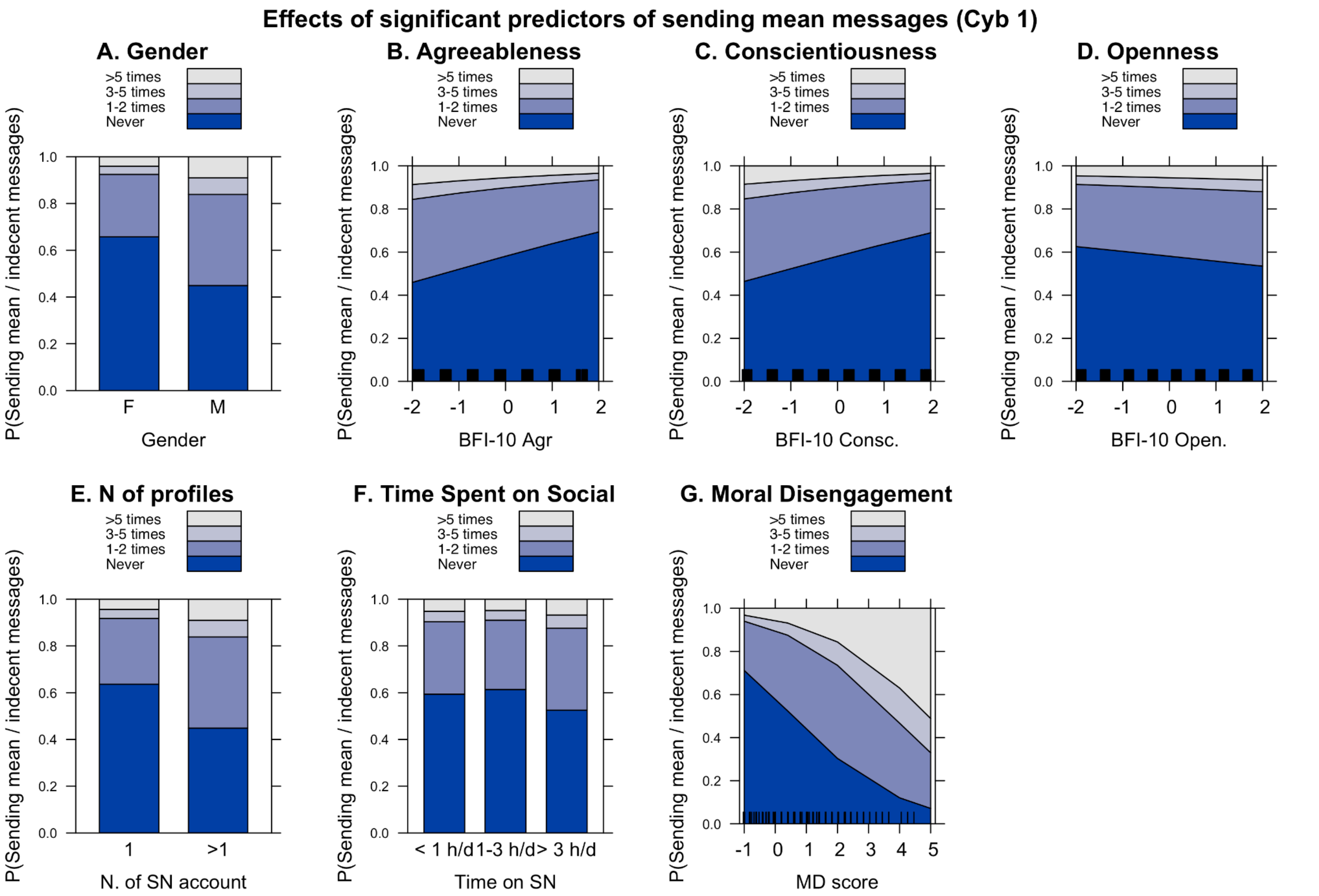

Effects of significant predictors of sending mean messages (Cyb 1)
A. Gender
B. Agreeableness
C. Conscientiousness
D. Openness
E. N of profiles
F. Time Spent on Social
G. Moral Disengagement
>5 times
3-5 times
1-2 times
Never
P(Sending mean / indecent messages)
1.0
0.8
0.6
0.4
0.2
0.0
F
M
Gender
-2
-1
0
1
2
BFI-10 Agr
BFI-10 Consc.
BFI-10 Open.
1
>1
N. of SN account
< 1 h/d
1-3 h/d
> 3 h/d
Time on SN
-1
0
1
2
3
4
5
MD score

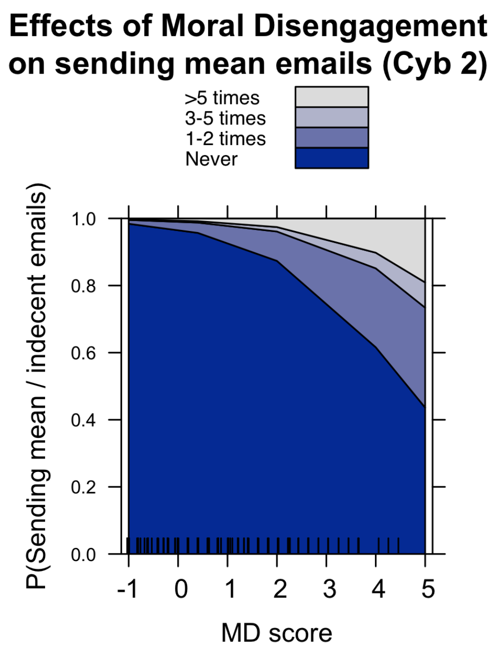
Effects of Moral Disengagement
on sending mean emails (Cyb 2)
>5 times
3-5 times
1-2 times
Never
P(Sending mean / indecent emails)
1.0
0.8
0.6
0.4
0.2
0.0
-1
0
1
2
3
4
5
MD score

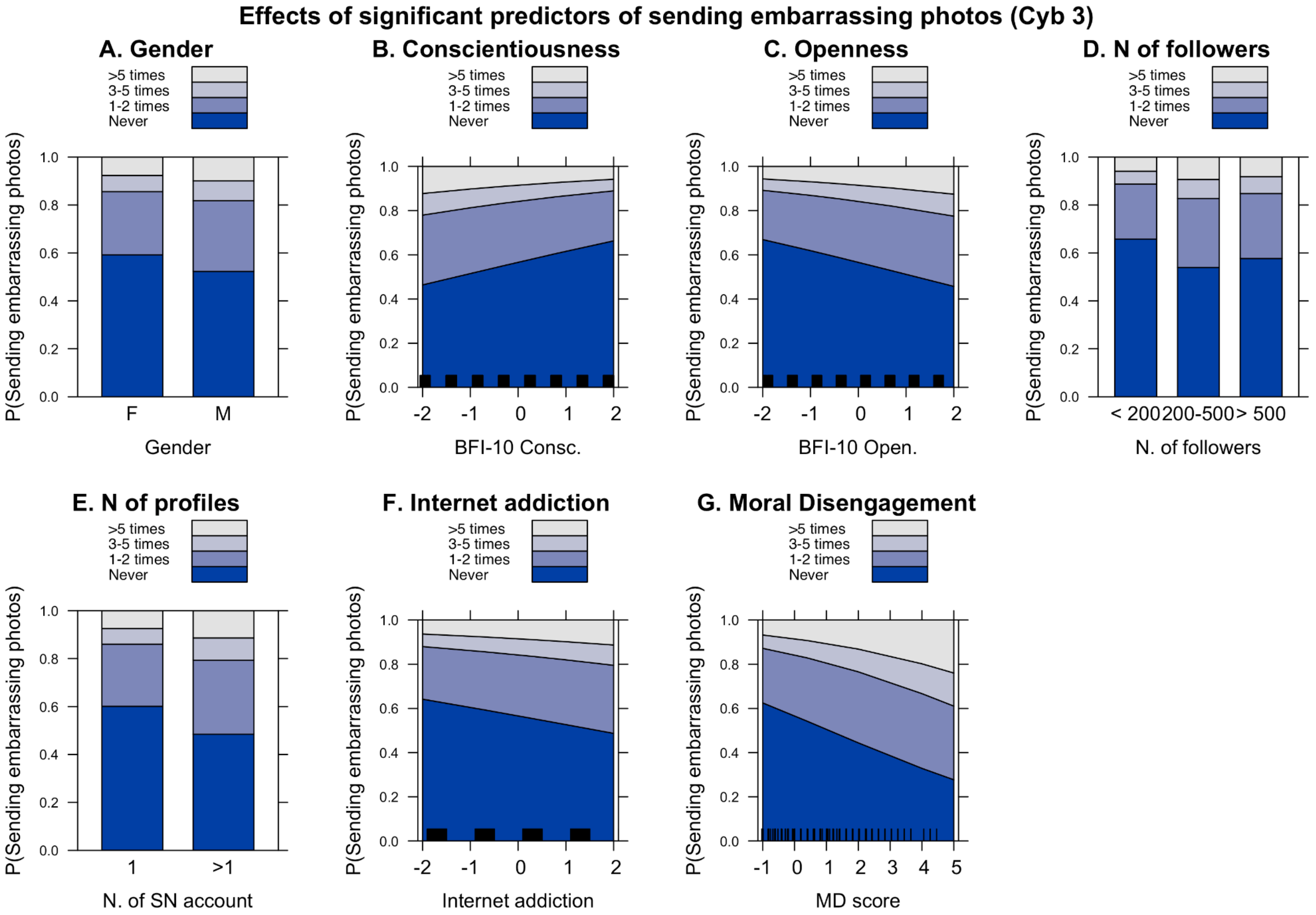

Effects of significant predictors of sending embarrassing photos (Cyb 3)
A. Gender
B. Conscientiousness
C. Openness
D. N of followers
E. N of profiles
F. Internet addiction
G. Moral Disengagement
>5 times
3-5 times
1-2 times
Never
P(Sending embarrassing photos)
Gender
F
M
BFI-10 Consc.
BFI-10 Open.
N. of followers
< 200
200-500
> 500
N. of SN account
1
>1
Internet addiction
MD score

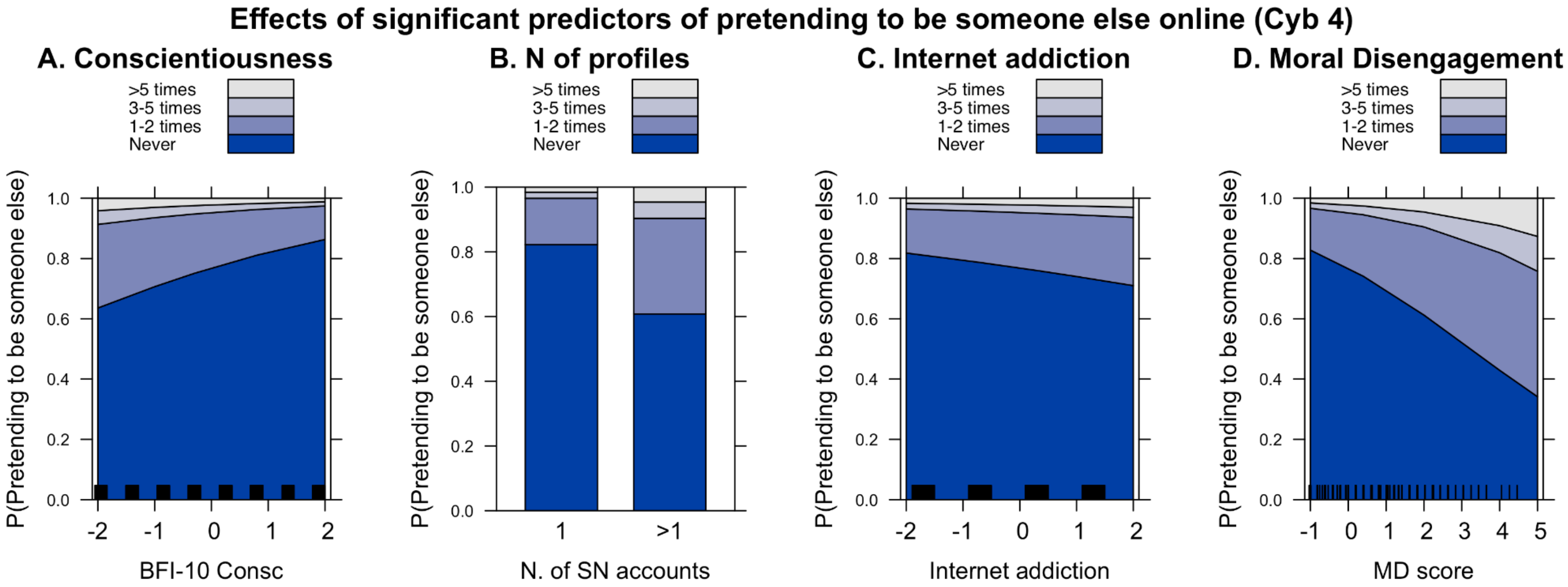

Effects of significant predictors of pretending to be someone else online (Cyb 4)
A. Conscientiousness
B. N of profiles
C. Internet addiction
D. Moral Disengagement
>5 times
3-5 times
1-2 times
Never
P(Pretending to be someone else)
BFI-10 Consc
N. of SN accounts
Internet addiction
MD score

Effects of significant predictors of revealing secrets (Cyb 5)

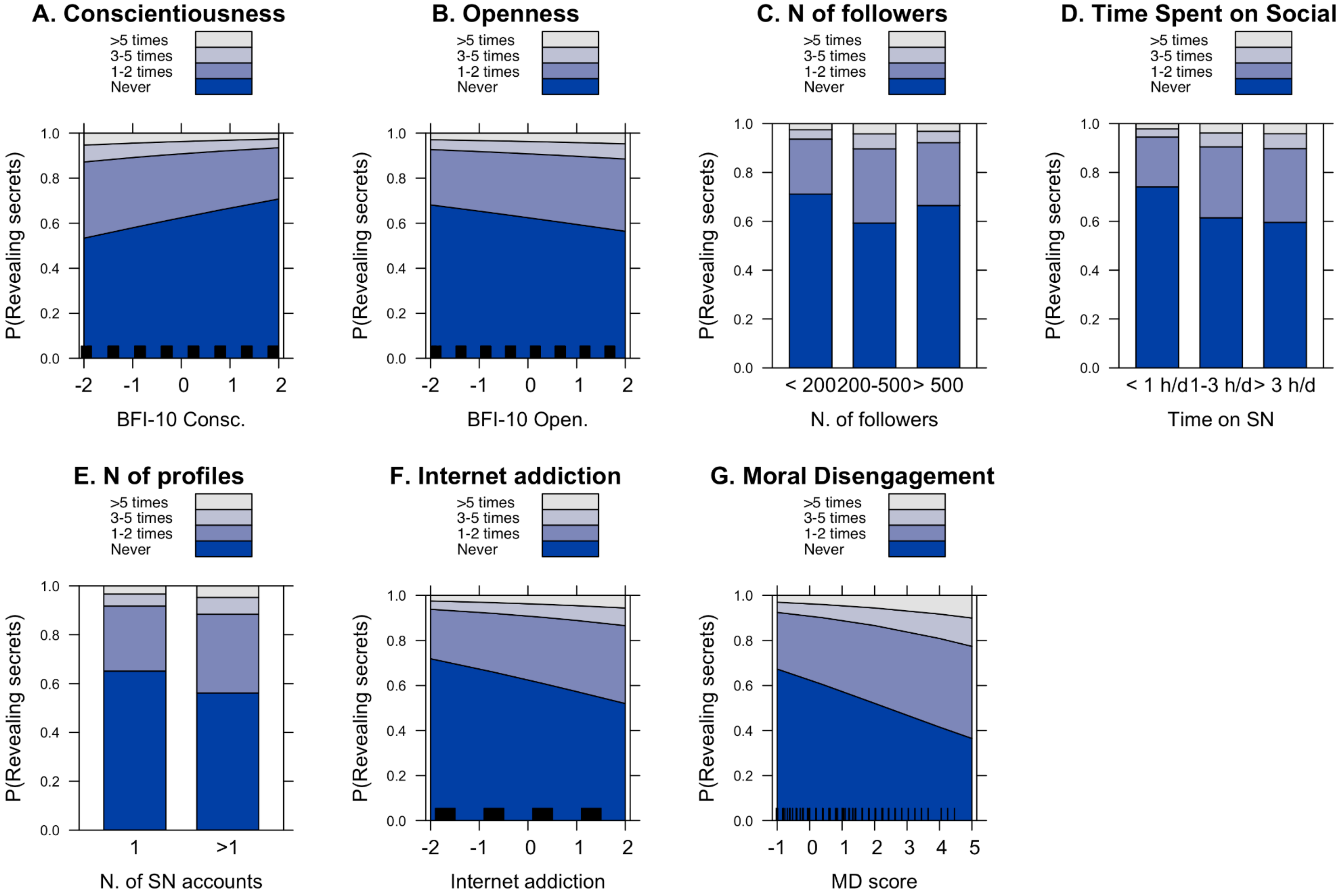

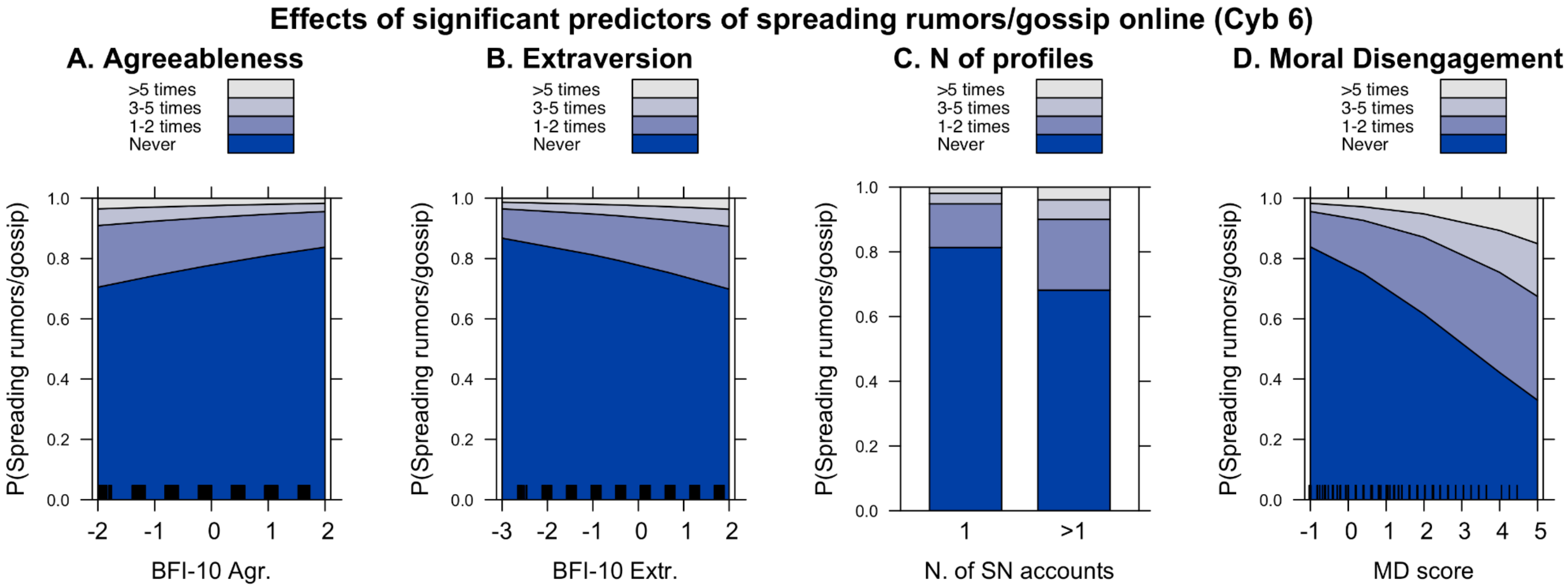

Effects of significant predictors of spreading rumors/gossip online (Cyb 6)
A. Agreeableness
B. Extraversion
C. N of profiles
D. Moral Disengagement
>5 times
3-5 times
1-2 times
Never
P(Spreading rumors/gossip)
1.0
0.8
0.6
0.4
0.2
0.0
-3
-2
-1
0
1
2
3
4
5
>1
BFI-10 Agr.
BFI-10 Extr.
N. of SN accounts
MD score
