# Supplementary material for: How many cyberbullying(s)? A non-unitary perspective for offensive online behaviours
Source: PLoS One. 2022 Jul 19;17(7):e0268838. doi: 10.1371/journal.pone.0268838 (PMC9295961; doi:10.1371/journal.pone.0268838)
Supplement: S2 File — The results of the multigroup analyses for three grouping factors are reported in this file: a) gender (females vs males), b) time on social network (>3 h/d vs 1–3 h/d), and number of social networks profiles (1 profile vs >1 profiles). For each factor we present the goodness-of-fit statistics of models assuming different levels of invariance and the path coefficients for the model assuming strong invariance. (PDF) [file pone.0268838.s009.pdf]

Supporting information 4: Results of the multigroups SEM analysis

In the following pages are presented the results of the multigroup SEM analyses conducted to investigate differences across groups defined by a) gender (females vs males), b) time on social network (>3 h/d vs 1-3 h/d), and number of social networks profiles (1 profile vs >1 profiles).

For each grouping factor we report the results of the measurement invariance tests, goodness of fit statistics of the models fit, and the diagram of the model assuming strong invariance, including the coefficients estimated for each group. All the code for reproducing the analyses is provided as S2 File.

Multigroup SEM analysis for Gender

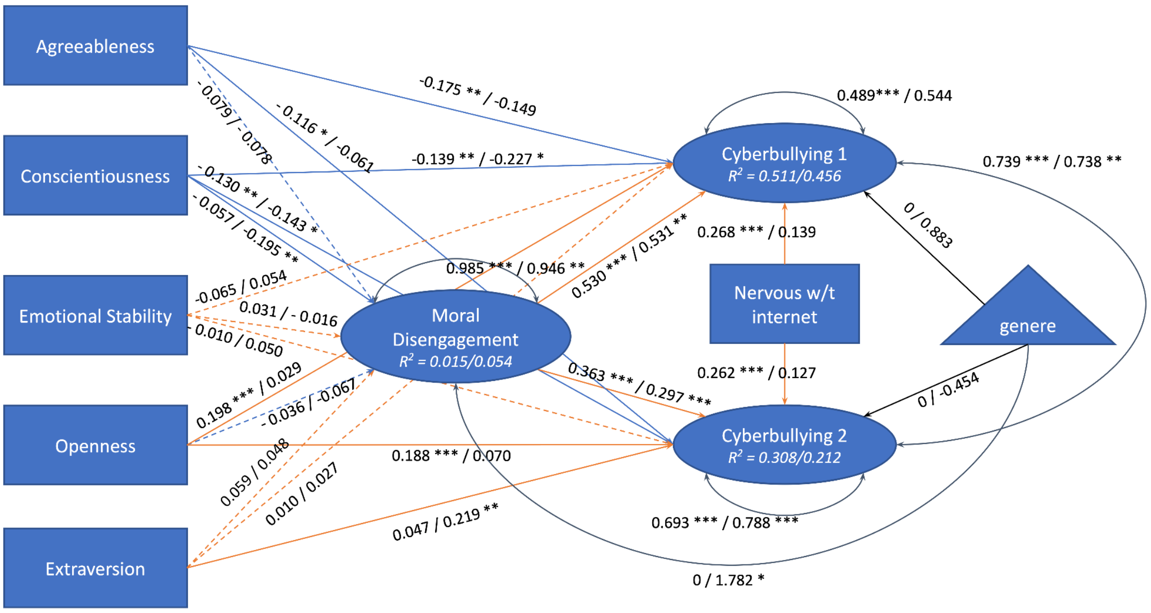

Figure S4.1 Path model and standardardized path coefficients for the multigroup SEM analysis by gender (female / male) assuming strong invariance. Solid lines represent cases in which for at least one of the groups the coefficient was significant, dashed lines represent cases in which for none of the groups the coefficient was significant. Blue lines represent negative associations and orange lines positive associations.

Table S4.1. Fit statistics of the multigroup SEM models testing different levels of invariance across gender, and chi-square test comparing nested models.

Invariance	*Constraints*	X^2	*df*	*CFI*	*MFI*	*RMSEA*	*srmr*	ΔX^2	*p*
Configural	factor structure	502.58	282	0.961	0.908	0.037	0.066		
Weak	loadings	535.56	293	0.957	0.899	0.038	0.069	18.40	0.072
Strong	loadings + intercepts	537.95	326	0.962	0.911	0.034	0.067	23.28	0.895

Table S4.1 legend. The top model in the table only assumes configural invariance across groups (same latent factors structure), and the other models add additional constraints fixing sets of parameters to be equal across groups (loadings and intercepts). In the model assuming weak invariance the factor loadings are constrained to be equal, and in the strong invariance model also the intercept of the indicators of the latent variables. A non-significant change in the value of Chi-square was taken as evidence that the more restrictive model (higher invariance) fitted data as well as the less restrictive model, and therefore should be preferred (being more parsimonious).

Multigroup SEM analysis for Time on Social Networks

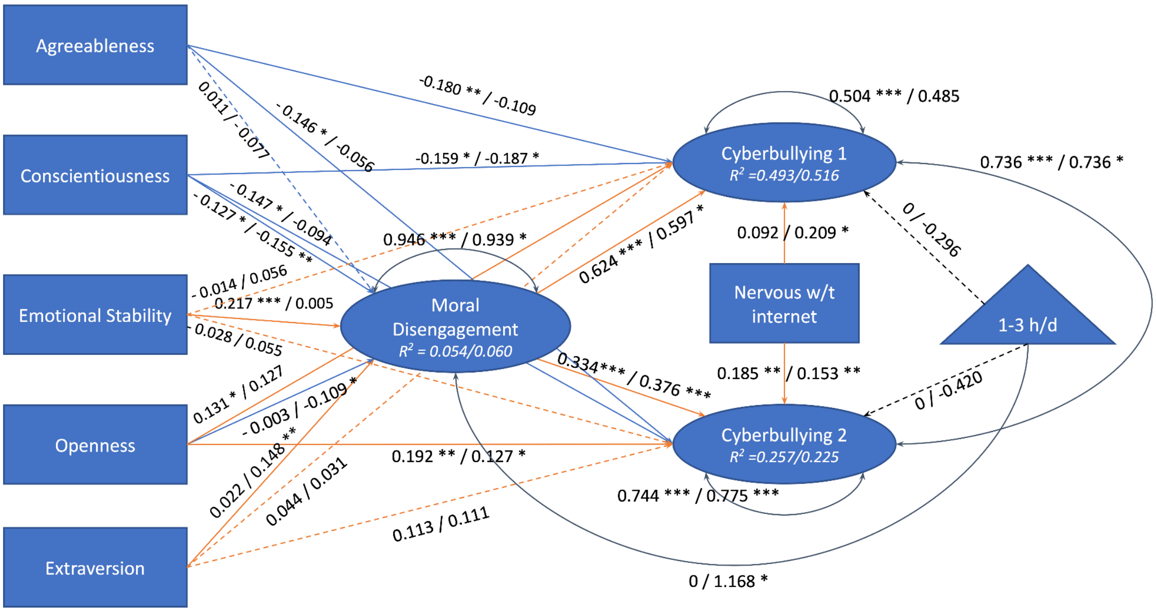

Figure S4.2 Path model and standardardized path coefficients or the multigroup SEM analysis by time on social networks (>3 h/d / 1-3 h/d) assuming strong invariance. Solid lines represent cases in which for at least one of the groups the coefficient was significant, dashed lines represent cases in which for none of the groups the coefficient was significant. Blue lines represent negative associations and orange lines positive associations.

Table S4.2. Fit statistics of the multigroup SEM models testing different levels of invariance across time on social networks, and chi-square test comparing nested models.

Invariance	*Constraints*	X^2	*df*	*CFI*	*MFI*	*RMSEA*	*srmr*	ΔX^2	*p*
Configural	factor structure	415.63	282	0.979	0.935	0.031	0.061		
Weak	loadings	432.59	293	0.978	0.933	0.031	0.063	9.74	0.554
Strong	loadings + intercepts	442.41	326	0.981	0.944	0.027	0.062	41.58	0.145

Table S4.2 legend. The top model in the table only assumes configural invariance across groups (same latent factors structure), and the other models add additional constraints fixing sets of parameters to be equal across groups (loadings and intercepts). In the model assuming weak invariance the factor loadings are constrained to be equal, and in the strong invariance model also the intercept of the indicators of the latent variables. A non-significant change in the value of Chi-square was taken as evidence that the more restrictive model (higher invariance) fitted data as well as the less restrictive model, and therefore should be preferred (being more parsimonious).

Multigroup SEM analysis for N. of SN profiles

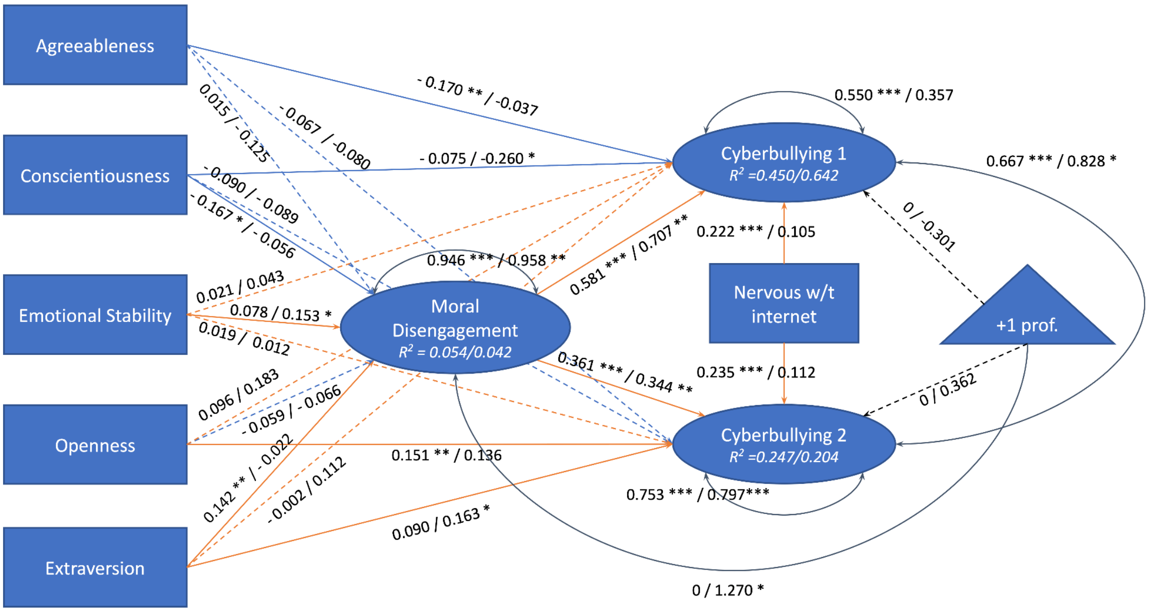

Figure S4.3 Path model and standardardized path coefficients for the multigroup SEM analysis by the number of SN profiles (1 profile / >1 profile) assuming strong invariance. Solid lines represent cases in which for at least one of the groups the coefficient was significant, dashed lines represent cases in which for none of the groups the coefficient was significant. Blue lines represent negative associations and orange lines positive associations.

Table S4.3. Fit statistics of the multigroup SEM models testing different levels of invariance by number of SN profiles, and chi-square test comparing nested models.

Invariance	*Constraints*	X^2	*df*	*CFI*	*MFI*	*RMSEA*	*srmr*	ΔX^2	*p*
Configural	factor structure	444.12	282	0.974	0.922	0.034	0.062		
Weak	loadings	467.93	293	0.972	0.916	0.035	0.064	12.22	0.347
Strong	loadings + intercepts	472.19	326	0.976	0.930	0.030	0.062	25.09	0.837

Table S4.3 legend. The top model in the table only assumes configural invariance across groups (same latent factors structure), and the other models add additional constraints fixing sets of parameters to be equal across groups (loadings and intercepts). In the model assuming weak invariance the factor loadings are constrained to be equal, and in the strong invariance model also the intercept of the indicators of the latent variables. A non-significant change in the value of Chi-square was taken as evidence that the more restrictive model (higher invariance) fitted data as well as the less restrictive model, and therefore should be preferred (being more parsimonious).
