# Supplementary material for: How many cyberbullying(s)? A non-unitary perspective for offensive online behaviours
Source: PLoS One. 2022 Jul 19;17(7):e0268838. doi: 10.1371/journal.pone.0268838 (PMC9295961; doi:10.1371/journal.pone.0268838)
Supplement: Attachment — Submitted filename: How many cyberbullying(s).docx [file pone.0268838.s010.docx]

**"How many cyberbullying(s)? A non-unitary perspective for offensive online behaviours"**

Dear author and editor,

I consider this research to be rigorous and useful to the scientific community.

However, we should improve some aspects to favor the reader's understanding. These are minor revisions.

Abstract: add all sociodemographic data, sex and age.

Theoretical framework: add more meta-analysis references that support your theoretical framework. Here are some studies that could be interesting:

Pan, Y. C., Chiu, Y. C., & Lin, Y. H. (2020). Systematic review and meta-analysis of epidemiology of internet addiction. Neuroscience & Biobehavioral Reviews, 118, 612-622. https://doi.org/10.1016/j.neubiorev.2020.08.013.

Modecki, K. L., Minchin, J., Harbaugh, A. G., Guerra, N. G., & Runions, K. C. (2014). Bullying Prevalence Across Contexts: A Meta-analysis Measuring Cyber and Traditional Bullying. Journal of Adolescent Health, 55(5), 602-611. https://doi.org/10.1016/j.jadohealth.2014.06.007

Marciano, L., Schlz, P. J., & Camerini, A. L. (2020). Cyberbullying perpetration and victimization in youth: A meta-analysis of longitudinal studies. Journal of Computer-Mediated Communication, 25(2), 163-181. https://bit.ly/3iK8b0g

Lei, H., Li, S., Chiu, M. M., & Lu, M. (2018). Social support and Internet addiction among mainland Chinese teenagers and young adults: A meta-analysis. Computers in Human Behavior, 85, 200-209. https://doi.org/10.1016/j.chb.2018.03.041. https://doi.org/10.1016/j.chb.2018.03.041

Lozano-Blasco, R., Cort'es-Pascual, A., & Latorre-Martínez, M. P. (2020). Being a cybervictim and a cyberbully - The duality of cyberbullying: A meta-analysis. Computers in Human Behavior, 111, 106444. https://doi.org/10.1016/j. chb.2020.106444.

1.	Lozano-Blasco, R.; Cortés-Pascual, A. Problematic Internet uses and depression in adolescents: A meta-Analysis. Comunicar 2020, 28, 109-120. [Google Scholar] [CrossRef].

Holt, M. K., Vivolo-Kantor, A. M., Polanin, J. R., Holland, K. M., DeGue, S., Matjasko, J. L., Wolfe, M., & Reid, G. (2015). Bullying and Suicidal Ideation and Behaviors: A Meta-Analysis. PEDIATRICS, 135(2), e496-e509. https://doi.org/10.1542/peds.2014-1864.

Methodology: add a main figure where the procedure and research design are visually explained.

Participants: write the sociodemographic information in a table to make it more visual. Clarify the regions that have been involved in Italy, a heat map would bring innovation and creativity.

Instruments: very well developed but do not forget to put the Italian version next to the name. In the references it is fine, but it is important for the scientific community to see that you have used standardized tests adapted to their culture.

Statistical analysis: very well developed, do you think that mediation analysis could provide more information on the moderating variables?

Results: very interesting but Figure 1 has poor visual quality. The concepts are very good but it looks very blurry and the size of the numbers is very small. The same happens with figure 2. Try to use some application like Canva, Flourish that allows you to improve the quality.

Discussion: I liked it very much, but to give it a plus of quality I recommend that you review more references on the use of social networks and cyberbullying. Add more meta-analysis studies that allow you to give more robustness to your assertions. Adding the hypotheses in the text was a great idea. The limitations are very sincere and clear, but I miss two paragraphs on practical implications this research would have.

Conclusions: they are great, but you usually don't put references in this section, could you upload those references to discussion and rewrite those sentences in a more general way?

Supplementary material: it is very honest that you upload the questionnaire, but it is an opportunity for this to be used by others. I would encourage you to upload the data of your rating along with it, so that it can be used.

Once again, thank you for your thoroughness and good work.
